# Structural Implications of STAT3 and STAT5 SH2 Domain Mutations

**DOI:** 10.3390/cancers11111757

**Published:** 2019-11-08

**Authors:** Elvin D. de Araujo, Anna Orlova, Heidi A. Neubauer, Dávid Bajusz, Hyuk-Soo Seo, Sirano Dhe-Paganon, György M. Keserű, Richard Moriggl, Patrick T. Gunning

**Affiliations:** 1 Centre for Medicinal Chemistry, University of Toronto at Mississauga, Mississauga, ON L5L 1C6, Canada; e.dearaujo@mail.utoronto.ca; 2 Department of Chemical & Physical Sciences, University of Toronto at Mississauga, Mississauga, ON L5L 1C6, Canada; 3 Institute of Animal Breeding and Genetics, University of Veterinary Medicine, A-1210 Vienna, Austria; Anna.Orlova@vetmeduni.ac.at (A.O.); Heidi.Neubauer@vetmeduni.ac.at (H.A.N.); Richard.Moriggl@vetmeduni.ac.at (R.M.); 4 Medicinal Chemistry Research Group, Research Center for Natural Sciences, 1117 Budapest, Hungary; bajusz.david@ttk.mta.hu (D.B.); keseru.gyorgy@ttk.mta.hu (G.M.K.); 5 Department of Cancer Biology, Dana-Farber Cancer Institute, Department of Biological Chemistry & Molecular Pharmacology, Harvard Medical School, Boston, MA 02115, USA; hux@crystal.harvard.edu (H.-S.S.); dhepag@crystal.harvard.edu (S.D.-P.); 6 Department of Biological Chemistry, Department of Biological Chemistry & Molecular Pharmacology, Harvard Medical School, Boston, MA 02115, USA

**Keywords:** STAT3, STAT5, SH2 domain, mutations, cancer, autosomal-dominant hyper IgE syndrome, inflammatory hepatocellular adenomas, T-cell large granular lymphocytic leukemia, T-cell prolymphocytic leukemia, growth hormone insensitivity syndrome

## Abstract

Src Homology 2 (SH2) domains arose within metazoan signaling pathways and are involved in protein regulation of multiple pleiotropic cascades. In signal transducer and activator of transcription (STAT) proteins, SH2 domain interactions are critical for molecular activation and nuclear accumulation of phosphorylated STAT dimers to drive transcription. Sequencing analysis of patient samples has revealed the SH2 domain as a hotspot in the mutational landscape of STAT proteins although the functional impact for the vast majority of these mutations remains poorly characterized. Despite several well resolved structures for SH2 domain-containing proteins, structural data regarding the distinctive STAT-type SH2 domain is limited. Here, we review the unique features of STAT-type SH2 domains in the context of all currently reported STAT3 and STAT5 SH2 domain clinical mutations. The genetic volatility of specific regions in the SH2 domain can result in either activating or deactivating mutations at the same site in the domain, underscoring the delicate evolutionary balance of wild type STAT structural motifs in maintaining precise levels of cellular activity. Understanding the molecular and biophysical impact of these disease-associated mutations can uncover convergent mechanisms of action for mutations localized within the STAT SH2 domain to facilitate the development of targeted therapeutic interventions.

## 1. Introduction

Several key cellular pathways converge on the multidomain signal transducer and activator of transcription (STAT) proteins highlighting their importance in the development and progression of oncogenic and malignant diseases. Conventional STAT activation is initiated by cytokine or growth-factor interactions with extracellular receptors, stimulating SH2 domain-mediated recruitment of tyrosine kinases and STAT isoforms to the receptor cytoplasmic domains [1,2]. Nuclear translocation and accumulation of the resulting phosphorylated STAT dimers facilitates transcription of a wide array of gene products involved in proliferation and cellular survival including C-MYC [3], BCL-XL [3], MCL-1 [4], FOXP3 [5], BCL-2 [6], HIF [7], D-type cyclins [8], IGF-1 [9], and self-regulation of STAT3/STAT5 [10]. Normal STAT function is dependent on the SH2 domain which arbitrates homo- or hetero- STAT dimerization as well as multiple protein–protein interactions. As such, structurally altered SH2 domains exhibit considerable effects on STAT activity, leading to either hyperactivated or refractory STAT mutants. These critical roles in governing the transcriptional capacity, coupled with the relatively shallow binding surfaces elsewhere on the protein, resulted in the STAT SH2 domain dominating therapeutic interest for small molecule inhibitor development and intervention [11,12,13,14,15]. However, currently there are no clinical drug candidates directly targeting the STAT protein family. This is partially due to the limited structural data available on the STAT SH2 domains or their mutated disease-associated counterparts, and further compounded by observations that STAT SH2 domains are distinct from those found in other well characterized systems such as Src kinase. Here, we summarize structural features of STAT-type SH2 domains in the context of STAT3/STAT5 disease-associated mutations, and discuss their effects on protein activity, as well as potential new druggable pockets within the STAT SH2 domain.

## 2. Structure of STAT SH2 Domains

SH2 domains are modular units that arose within multicellular life, approximately 600 million years ago, and are therefore heavily tied to metazoan signal transduction [16]. There are 121 human SH2 domains that are classified into different groups based on their structural or phylogenetic characteristics [16]. Broadly, they have been classified into either STAT- or Src-type SH2 domains based on the presence of either an α-helix (STAT-type) or β-sheet (Src-type) at the C-terminus [17]. Alternatively, phylogenetic analysis has categorized SH2 domain-containing proteins into 38 different sub-families [16]. Functional activity-based screens have also been employed to stratify SH2 domain-containing proteins into four categories based on the identity of the fifth residue in the βD strand, which has been shown to be a critical determinant in phospho-peptide selectivity [18,19]. Despite different methods for classification, all SH2 domains contain conserved structural motifs that are canonical to the core function of phospho-Tyr (pY) peptide binding. These features represent an evolutionary compromise to preserving critical structural motifs while maintaining highly specific peptide recognition capacity.

**Figure 1:**
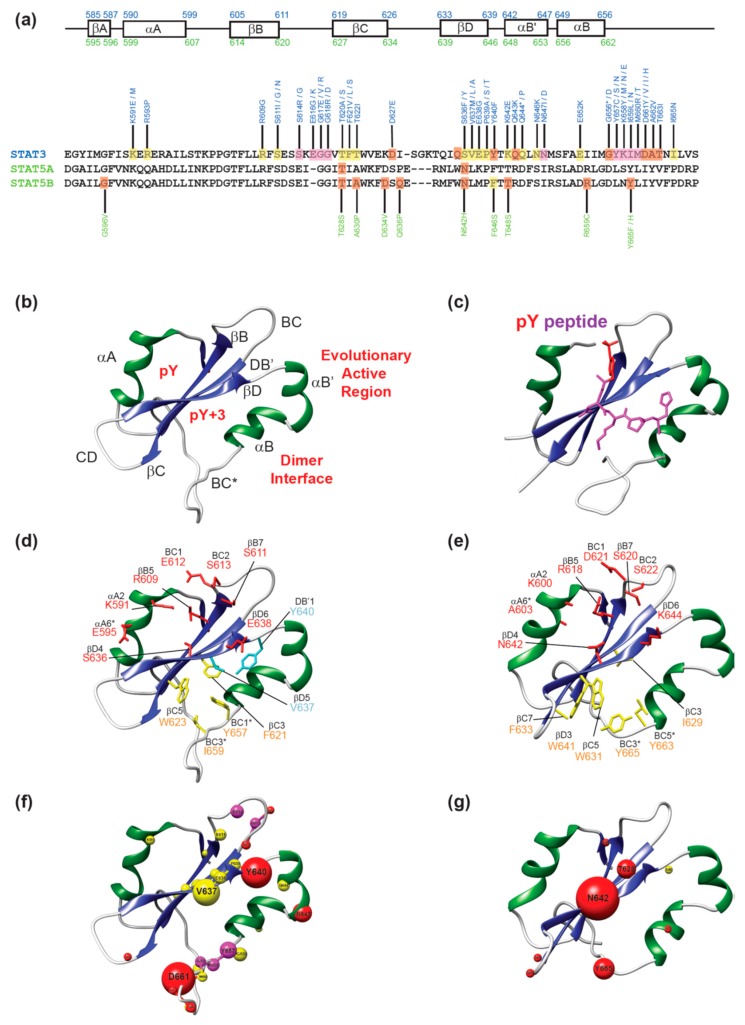
(**a**) Secondary structural motifs in STAT3 (blue) and STAT5 (green) with mutations annotated; (**b**) Structure of STAT3 SH2 domain; (**c**) Structure of pY-peptide-STAT1 SH2 domain. The pY residue is depicted in red with the C-terminal residues in violet; (**d**) Structure of STAT3 SH2 domain with Sheinerman residues (red), hydrophobic system residues (yellow) and the selectivity filter (cyan); (**e**) Structure of STAT5B SH2 domain with the same color scheme as above; (**f**) STAT3 SH2 domain with all mutations highlighted in spheres. The volume of each sphere is proportional to frequency of cases identified. Red spheres indicate an activating mutation, yellow spheres indicate a destabilizing mutation and magenta spheres represent sites where both activating and refractory mutations are observed; (**g**) STAT5B SH2 domain with all mutations highlighted in spheres. The color scheme is the same as in (**f**). Protein structures were visualized using Chimera [21] with PDB codes: 4E68 [22] (STAT3), 6MBW (STAT5B) [23], 1YVL (STAT1) [24].

The structure of an SH2 domain consists of a central anti-parallel β-sheet (with the three β-strands conventionally labeled βB-βD) interposed between two α-helices (αA and αB), often referred to as the αβββα motif [16]. The structure and nomenclature for the motifs of STAT SH2 domains is shown in Figure 1a,b. The β-sheet partitions the SH2 domain into two subpockets, referred to as the pY (phosphate-binding) and pY+3 (specificity) pocket [16]. The pY pocket is formed by the αA helix, the BC loop (region connecting βB-βC strands) and one face of the central β-sheet. The pY+3 pocket is created by the opposite face of the β-sheet as well as residues from the αB helix and CD and BC* loops (regions connecting βC-βD strands and αB-αC helices, respectively). Both the pY and pY+3 pockets are common targets for drug design due to well defined features and conserved residues. Within the pY+3 pocket, there are additional clefts that also have drug targeting potential. This includes the C-terminal region of the pY+3 pocket, also known as the evolutionary active region (EAR) [17]. The EAR contains an additional α-helix (αB’) in STAT-type SH2 domains, as opposed to the Src-type which harbors a β-sheet (βE and βF although each strand is not always observed). Additionally, there is a cluster of non-polar residues (referred to as the hydrophobic system [20]) at the base of the pY+3 pocket that assists in stabilizing the conformation of the β-sheet and maintaining the integrity of the overall SH2 domain. The αB, αB’, and BC* loop also participate in SH2-mediated STAT dimerization forming important cross-domain interactions. Therefore, residues in the pY+3 pocket can have a dual effect on STAT dimerization capacity and phospho-peptide binding. Conventional phospho-peptide binding occurs perpendicular to the β-sheet with the peptide adopting a binding mode as illustrated with STAT1 in Figure 1c. The phospho-Tyr interacts with conserved amino acids in the pY pocket, while the C-terminal residues stretch across the SH2 domain into the pY+3 pocket.

These residues are critical for maintaining proper binding interactions to facilitate protein dimerization, and specific mutations here can alter normal STAT function. An issue of particular relevance for drug discovery is protein flexibility, and indeed, STAT SH2 domains exhibit a particularly flexible behavior even in sub-microsecond timescales [23,25]. Notably, the accessible volume of the pY pocket varies dramatically. Also, crystal structures do not necessarily preserve even the main, targetable pockets in an accessible state. This further underlines the importance of accounting for protein dynamics in STAT-directed drug discovery efforts.

## 3. Disease-Associated Mutations in STAT3 and STAT5B SH2 Domains

Sequencing analysis of patient samples has identified multiple point mutations within the SH2 domain of STAT3 (Table 1) and STAT5B (Table 2) leading to variable effects on physiological activity. In mice, homozygous disruption of STAT3 is embryonically lethal [26], and correspondingly germline homozygous loss-of-function (LOF) mutations have not been identified in humans. Heterozygous loss of STAT3 can be tolerated to different extents and contributes to immunological deficiencies, most commonly autosomal-dominant Hyper IgE syndrome (AD-HIES) as a result of a reduced STAT3-mediated Th17 T-cell response [27,28,29]. Classical STAT3 function is implicated in Th17 T-cell lineage commitment, through upregulation of RORγt, promoting the release of IL-17 and IL-22. This stimulates transcription of genes associated with Th17 development. Loss of STAT3 function strongly diminishes Th17 T-cell expansion, thereby reducing the immunologic response leading to recurrent staphylococcal infections and exceedingly high levels of IgE that contribute to clinical presentations of eczema and eosinophilia.

Comparatively, homozygous loss of both STAT5 gene products in mice is lethal late in embryonic development, due to the defective erythropoiesis and with loss of STAT5B manifesting with a myriad of physiological effects including sexual dimorphic body growth [104,105,106]. Clinical cases of patients with STAT5B LOF mutations on both alleles exhibit features similar to growth hormone insensitivity syndrome (GHIS). However, heterozygous human carriers of STAT5B LOF mutations generally do not present with any immunological deficiencies or growth complications, although there have been three germline dominant-negative heterozygous STAT5B mutations recently reported that result in postnatal growth impairment among other physiological symptoms [107]. Although these growth deficiencies are likely multifactorial in etiology, the growth hormone (GH)-growth hormone receptor (GHR) interactions that recruit JAK kinase and stimulate phosphorylation of STAT5B remain intact, suggesting a breakdown in corresponding STAT5B activity. STAT5B LOF patients often carry additional immunological burdens including reduced populations of several T-cell subtypes, suggesting multiple roles for STAT5 in T-cell differentiation [108].

Gain-of-function (GOF) germline mutations for STAT3 and STAT5 are rare and clinically diverse. STAT3 GOF mutations present with autoimmune responses, likely due to Th17 clonal expansion which also suppresses regulatory T-cell (Treg) formation. Clinical presentations of STAT3 GOF mutations also show parallels with STAT5 LOF mutations [28]. This is partially an effect of compensatory upregulation of SOCS3 (suppressor of cytokine signaling-3) which strongly inhibits hyperactivated STAT3, but by extension also dampens STAT5 and STAT1 activity. This potently reduces basal levels of STAT5 leading to growth immunodeficiencies.

Contrastingly, multiple de novo somatic GOF mutations arise in STAT3 and STAT5B leading to cancer pathogenesis, and such mutations have been implicated in both solid and liquid tumors. Hyperactivated STAT3 is identified in patients with diverse phenotypes and multiple somatic mutations have been associated with T-LGLL (T-cell large granular lymphocytic leukemia, 40–70% of all cases) and NK-LGLL (natural killer cell large granular lymphocytic leukemia, 30% of all cases), as well as different hepatocellular adenomas [40]. STAT5 is upregulated, directly and indirectly, through multiple mechanisms in several hematological malignancies leading to neoplastic transformation. For instance, in ~30% of acute myeloid leukemia (AML) cases, STAT5B is activated by mutated FLT3 (Fms-Like Tyrosine Kinase 3) [109]. Similarly, ~99% of all chronic myeloid leukemia (CML) cases, which result from the appearance of the Philadelphia chromosome, result in STAT5B hyperactivation [110]. B-cell cancers, such as B-ALL (B-cell acute lymphoid leukemia) and B-CLL (B-cell chronic lymphoid leukemia) are driven by upregulation of IL-7 and IL-22 which also simulate STAT5B [111,112,113]. Acute and chronic T-cell cancers have lower incidence, but a larger diversity, and are heavily implicated by STAT5B GOF driver mutations [84,114,115]. STAT GOF mutations generally stabilize protein structure thereby enhancing transcriptional output, leading to apoptosis-evading and survival phenotypes. LOF mutants generally distort secondary structure and contribute to either loss of activity or increased STAT degradation. Herein, we highlight the structural significance and mechanism of action of malignant STAT3/5B point mutations in the SH2 domain. Notably, STAT5A mutations are less frequently identified which is likely due to the differential roles of the protein isoforms. Although STAT5B has been characterized as a driver in several malignancies, STAT5A has been associated with tumor suppression [116,117]. STAT5A and STAT5B have similar SH2 domains (~93% amino acid similarity) with significant changes in the βD strand which likely contributes to varying peptide selectivity.

### 3.1. Mutations in the pY Pocket

As previously described, the pY pocket is formed by the αA helix, BC loop, and one face of the central β-sheet, and it harbors an overall positive electrostatic potential to stabilize binding with the electronegative phospho-Tyr side-chain (Figure 1). This region of the SH2 domain is characterized by strongly conserved residues that facilitate interactions with the phosphorylated peptide. It includes the SH2 domain signature sequence as well as a group of 8 phospho-Tyr interacting amino acids that have been collectively referred to as Sheinerman residues [20]. The SH2 domain signature sequence, FLXRXS (where X is a hydrophobic amino acid), corresponds to FLLRFS in all STAT proteins and is located on the βB strand. The eight Sheinerman residues correspond to the positions: αA2, αA6, βB5, βB7, BC1, BC2, βD4 and βD6 [20]. Critically, the βB5 residue is located within the SH2 domain signature sequence as an invariant Arg residue, which is conserved in 118 of 121 SH2 domain-containing proteins [16]. This indispensable Arg residue is the principal binding partner for phospho-Tyr with the side-chain guanidinium group participating in a bidentate ionic interaction with the phosphate. The side-chains of the αA2 (Arg/Lys in 118/121 SH2 domains), βB7 (Ser in 106/121 SH2 domains) and βD4 (His in 80/121 SH2 domains) residues also participate in direct interactions with the phospho-Tyr [16]. Notably, the αA2, βB7, and βD4 residues correspond to Lys591, Glu638, and Ser636 in STAT3 and Lys600, Ser620, and Asn642 in STAT5B. These interactions are highly important for phospho-peptide binding and are reported to contribute to >50% of Gibbs free energy of the protein–peptide interaction [16]. The high fidelity of these interactions ostensibly suggests that a mutation at these sites will have a dramatic effect on the activity or binding capacity of STAT3/5.

#### 3.1.1. Mutations in Sheinerman Residues

Mutations identified at the αA2 site in STAT3 (Lys591Glu [30] and Lys591Met [31]) contribute to AD-HIES, a STAT3-deficient malignancy, due to removal of the positively-polarized Lys side-chain that directly coordinates the phospho-Tyr. Similarly, mutations at other critical residues in the pY pocket present with analogous clinical outcomes. Mutation of the invariant βB5 position (Arg609Gly [33]) in STAT3 leads to AD-HIES, presenting with reduced expression profiles of Th17 T-cells and high serum levels of IgE (11,300 IU/mL). Replacement of βD4 (Ser636Phe [49]) leads to strongly elevated IgE levels (17,407 IU/mL) with presentation of eczema, abscesses, and pneumonia, also characteristic of AD-HIES pathologies. Interestingly, a patient with a semi-conservative substitution (Ser636Tyr [30]) that retains H-bonding capacity at the βD4 position still presented with a reduced percentage of Th17 cells (0.31%) compared to healthy patients (>1%), highlighting the substantive role of changes in sterics at the phosphate binding positions. Examining the prevalence of mutations within the remaining Sheinerman residues shows infrequent mutations, such as STAT3 βB7 (in which Ser611Gly [34]/Asn [36] have been reported), which also lead to ablation of activity and AD-HIES. In cellulo studies with Ser611Asn indicate a reduced capacity for activation by phosphorylation consistent with altered phospho-Tyr binding [118]. Similarly, substitution at the Sheinerman βD6 position is also capable of triggering the AD-HIES phenotype. Notably, this βD6 (Glu638Gly [49,51]) mutation likely causes gross conformational changes in the β-sheet due to removal of a complete side-chain. Although mutations in the pY pocket of STAT3 tend to abolish peptide binding, specific mutations in the BC loop lead to either hyperactivation and LGL leukemias or protein dysfunction and AD-HIES. Since the BC loop is directly involved in multiple domain interactions including the pY and pY+3 pockets, mutations identified in this region will be discussed in detail in Section 3.3.

In STAT5, mutations at the conserved Sheinerman residues have not been identified apart from the βD4 position. In the majority of SH2 domain-containing proteins, the βD4 residue is a His, directly coordinating the phosphate, and mutagenesis of this residue abolishes peptide binding capacity [16]. As seen with STAT3 (Ser636Tyr), modification in sterics at this position greatly modulates phospho-Tyr peptide binding. In STAT5B, the βD4 residue is an Asn642, and the absence of a conserved His residue may represent an evolutionary response to tune down the basal activity of STAT5. Notably, this residue is most frequently mutated in STAT5B (Asn642His) and has been reported in multiple cancer phenotypes (>150 cases [41,62,69,77,80,81,83,84,85,86,87,88,89,90,91,92,93,94,95,96,97,98,99,100,101,102]), most commonly T-cell-prolymphocytic leukemia (T-PLL), monomorphic epitheliotropic intestinal T-cell lymphoma (MEITL), and T-cell acute lymphoblastic leukemia (T-ALL). Asn642His is an extremely aggressive oncodriver of T-cell neoplasia and previous studies have shown multiple T-cell subset organ infiltration and transformation in transgenic mice [23,119]. Recently, the crystal structure for the STAT5B Asn642His mutation was reported which suggested different SH2 domain conformations with either a neatly packed βD strand forming a tight central β-sheet or a more dissociated βD strand that provides greater access to the SH2 domain [23]. Additionally, different biophysical studies [23,41] have confirmed the substantial increase (~5–7 fold) in the affinity of pY containing peptides for mutated STAT5B (Asn642His) compared to wild type. This provides a molecular basis for the lower threshold of mutant STAT5B towards cytokine activation and the aggressive phenotype observed in patients. The Asn642His mutation is also predicted to lead to a more stable dimer interface and reduced dephosphorylation kinetics, prolonging the lifetime of the activation state [23].

#### 3.1.2. Mutations Outside Sheinerman Residues

The mutational landscape of STAT3 and STAT5B within the pY pocket also extends to less conserved residues, predominantly on the βC strand. Mutations at βC4 have been identified in both STAT3 (Thr620Ser [33]/Ala [34,49]) and STAT5B (Thr628Ser). In STAT5B, this conservative mutation is commonly observed in T-PLL and T-ALL [86]. In vitro studies with STAT5B Asn642 variants have shown that bulkier substituents in the pY pocket reduce phospho-Tyr affinity [23]. Therefore, the loss of a single methylene group from Thr628 can better accommodate the cognate peptide yielding increased transcriptional activity. However, contrary effects are observed with Thr620Ser in STAT3, where this mutation, as well as Thr620Ala, both lead to reduced protein activity and AD-HIES. The contrasting effects of the same mutation at identical positions in STAT3 and STAT5B underscores the unique aspects of each structural motif, and more broadly, the potential for isoform specific drug targeting. Generally, STAT3 is less robust to molecular modifications with slight changes capable of strongly diminishing activity. This is unusual considering the melting temperature of isolated STAT3 (52.5 ± 0.7 °C) is higher than STAT5B (44.5 ± 0.3 °C) [120]. The total protein stability is likely the result of both structural and complex protein dynamics and requires further investigation. One destabilizing mutation has been observed in the pY pocket of STAT5B at the βC4 position (Ala630Pro [121]) which disrupts the β-sheet, reducing protein solubility and leading to misfolding and a clinical presentation of severe growth deficiency. Thus, destabilizing mutations are observed in STAT5B, but with a reduced frequency.

### 3.2. Mutations in the pY+3 Pocket

Adjacent to the pY pocket of the SH2 domain is the pY+3, or specificity pocket which interacts with C-terminal residues of the phospho-Tyr peptide. The βD strand is critical in facilitating these interactions, particularly the βD5 residue which controls accessibility to this pocket. In Src-type SH2 domains, additional interactions with the cognate phospho-peptide occur between residues in the evolutionary active region (EAR). This includes the βE and βF strands as well as the loops in between these structural elements. In STAT3 and STAT5, the EAR motif is comprised of an α-helix (αB’), and the corresponding interactions occur within the DB’ region (loop in between βD strand and αB’ helix), αB’, and αB helices. There is also a clustering of predominantly aromatic, hydrophobic residues at the base of the pY+3 pocket which has been referred to as the hydrophobic system (βC3, βC5, βD3, BC1*, and BC3*). In STAT3, this includes residues Phe621 (βC3), Trp623 (βC5), Tyr657 (BC1*), and Ile659 (BC3*) and in STAT5B, Ile629 (βC3), Trp631 (βC5), Phe633 (βC7), Trp641 (βD3), Leu663 (BC1*) and Tyr665 (BC3*). In STAT3, the hydrophobicity of this pocket is reduced by the presence of Gln635 and Lys626 at the βD3 and βC7 positions, respectively. The increased polarity leads to a reduced STAT3 preference for phospho-peptides with non-polar residues in the C-terminal positions compared to STAT5B. The SH2 dimerization interface is in close proximity to the pY+3 pocket and is formed by the BC* loop, αB’ helix and one face of the αB helix. As such, alterations in the pY+3 pocket directly affect the dimerization interface.

#### 3.2.1. Mutations in the Hydrophobic System and βD Strand

The pY+3 pocket is a hotspot for STAT SH2 domain mutations. Generally, mutations that increase the polarity of this pocket result in protein destabilization and LOF. This was demonstrated in Epstein–Barr virus (EBV)-transformed B-cells expressing AD-HIES-associated STAT3 mutations. In these assays, the half-life of wild type STAT3 (25 ± 2 h) was shown to be substantially reduced by polar mutations at βD5 (Val637Met = 5.3 ± 4.5 h) and BC1* (Tyr657Cys = 5.5 ± 4.1 h) [122]. Conversely, STAT3 mutant Tyr640Phe (DB’1), which is a commonly identified mutation in solid and liquid tumors, leads to constitutive activation across several cell lines (hepatic epithelial cells, lung carcinomas, fibroblasts, etc.) through enhanced stability of STAT3 dimerization, nuclear accumulation and increased transcriptional activity following IFNγ stimulation [44,47,48,59,60,61,62,65,66,67,68,69]. In wild type STAT3, Tyr640 points directly into the hydrophobic system. Increasing hydrophobicity, through removal of the hydroxyl group tightens the packing of the pocket and enhances the activation potential. The STAT3 Tyr640Phe mutation has been identified in over >110 cancer cases in the COSMIC database and is most frequently observed in patients with T-LGLL. An analogous mutation is observed at the BC3* site in STAT5B where the second most frequent mutation (Tyr665Phe) results in STAT5B hyperactivation and has also been observed in patients with T-LGLL.

Within the hydrophobic system, the presence of aromaticity for π-π stacking interactions from key residue side-chains is strongly favored over non-aromatic Van der Waals interactions. This is especially seen at the βC3 position with patients harboring Phe621Val [36,37,50]/Leu [51,52]/Ser [53] mutations resulting in STAT3-deficient AD-HIES. In cellulo mutagenesis studies with the Phe621Val mutation have elucidated impaired STAT3 phosphorylation, resulting in defective DNA binding capacity. This strong requirement for aromaticity is also observed in STAT5B, where Tyr665His leads to a hyperactive mutant, since it retains the aromaticity of the imidazole ring to interact with Trp631 (βC5), despite the increase in side-chain polarity.

In addition to the hydrophobic system, the βD strand is critical for controlling pocket accessibility. This is primarily governed by the residue at the βD5 position in SH2 domains and corresponds to Val637 in STAT3. This residue serves as a selectivity filter, interacting with C-terminal amino acids of the phospho-Tyr peptide. In Src kinase, the βD5 residue corresponds to a Tyr and the aromatic ring is sandwiched between the Glu (pY+1 residue) and Ile (pY+3 residue) of the phospho-peptide [18]. In other SH2 domain-containing kinases, the βD5 residue interacts with all three C-terminal amino acids of the pY-peptide. Given the critical nature of this site, it is not unexpected that mutations strongly impair STAT3 activity. STAT3 Val637Met has been identified in a number of patient samples (>40 cases) and is associated with AD-HIES due to impaired response to cytokine activation and transcriptional activity. The importance of Val637Met [30,33,34,36,49,56] is further underscored by insensitivity to 100-fold increases in IL-6 to simulate phosphorylation. This inability to recognize specific phospho-peptides contributes to the defective STAT pathway observed in AD-HIES. As a crucial selectivity filter, even semi-conservative mutations Val637Leu [36] and Val637Ala [30] have also been shown to be disruptive to STAT3 activity and result in AD-HIES. Although STAT3 Val637Met may be a result of reduced protein stability [122], circular dichroism spectra for STAT3 Val637Ala suggest that this substitution does not cause large structural perturbations. Alternatively, this substitution likely reduces phospho-peptide binding in the pY+3 pocket [123]. The βD7 position also assists in the orientation of the βD5 residue, and mutation of the rigid Pro639 to 639Ala [37]/Ser [30,34]/Thr [35] also results in AD-HIES.

#### 3.2.2. Mutations in the Dimerization Interface

In comparison to pY and pY+3 pockets of the SH2 domain, the dimerization interface represents a delicate balance in carefully regulating STAT activity. This region is littered with disease-causing mutations and slight changes to sterics or electronics at the αB, αB’, or BC* loop propagate their effects exponentially and lead to highly contrasting effects. There are multiple examples of such mutations throughout the dimerization interface. For instance, at the αB’6 site, patients with STAT3 Asn647Asp [36,72] exhibit symptoms of AD-HIES, but the Asn647Ile [57,65,67] mutation results in STAT3 hyperactivation manifesting as chronic lymphoproliferative disorder of NK cells (CPLD-NK) and T-LGLL. Comparable to trends observed at the central pY+3 pocket, hydrophobic or aromatic substitutions at the interface stabilize STAT3 dimer formation and subsequent phosphorylation, while changes in polarity, or in this case, an electrostatic reversal, effectively abolish STAT3 activity. Analogous effects are also observed at the BC1*–BC6* positions. At BC1* and BC2*, Tyr657Ser [74]/Asn [52]/Cys [30,34,36,58] and Lys658Glu [75] lead to AD-HIES, whereas Tyr657ins [47], Tyr657dup [48,72] and Lys658Met [67,72]/Asn [65]/Tyr [66] elicit several types of T-cell cancers [124]. At the BC3* position, Ile659Leu [57,72] has been characterized in T-LGLL, whereas the recently identified Ile659Asn [58] mutation distorts STAT3 activity leading to AD-HIES. Only destabilizing (AD-HIES causing) mutations have been identified at BC4* (Met660Arg [55] and Met660Thr [76]) in STAT3. The BC5* position was found to be genetically volatile with mutations occurring as Asp661His [65]/Val [78]/Tyr [41,47,48,60,63,65,67,68,72,77]/Ile [67], with all mutations resulting in STAT3 activation and enhanced response to cytokines. Finally, the BC6* (Ala662) and BC8* (Asn664) positions are critical SH2 domain interface determinants, where mutagenesis experiments have created artificial disulfide linked STAT3-Ala662Cys-Asn664Cys dimers that are constitutively active in cellulo and induce malignant transformation [66]. This further reinforces the role of the BC* loop in maintaining the dimer interface to control STAT activity. Individually, these Cys-mutations are likely destabilizing, but their pairing allows for covalent tethering of the STAT3 monomers and active dimer formation. Within STAT3, the disordered BC* loop tends to be the only site amenable to insertion/deletion mutations. Since different mutations in this BC* loop lead to either hyper- or refractory activity, likely the substitutions have some degree of compensatory effect that allow them to persist compared to other regions of the SH2 domain. Furthermore, the remaining motifs of the SH2 domain are highly structured and less likely to tolerate insertions or deletions.

The volatility shown by each of the residues in the BC* loop to trigger such extreme changes in STAT3 behavior underscores both the importance of this region to STAT activity, but also the necessity to understand the underlying molecular dynamics that result in such variability. This is particularly true for the BC5* residue which has no prescribed role in other SH2 domains. However, as seen above, its malignant capacity is revealed in >100 identified cases featuring a mosaic of mutations, according to the COSMIC database. Specifically, the STAT3 Asp661Tyr mutation represents one of the most frequently occurring mutation in the SH2 domain of STAT3 along with Tyr640Phe (>100 cases). Although the increases in hydrophobicity and aromaticity have been speculated as critical determinants for the aggressive nature of this mutation, the site-specific mechanism of activation remains unclear. Notably, both of these frequently cancer-associated mutation sites in STAT3 (Tyr640 and Asp661) are by default Phe and Ile respectively in STAT5B. This substitution to the STAT3 cancer-associated genotype in STAT5B suggests that the protein may be more optimized for protein dimerization. This is an interesting observation and further suggests a delicate evolutionary balance in STAT5B by potentially improving interactions at the dimerization interface while reducing activity through the lack of an efficient phosphate-coordinating βD4 residue (Asn642). The functional significance of these changes in STAT5B, compared to STAT3, has not been biophysically characterized and may also suggest that additional mechanisms are relevant to the disease-associated phenotype including changes to protein stability or transcriptional regulation.

### 3.3. Mutations in the Additional Regions of the SH2 Domain

Mutational hotspots in regions outside the pY and pY+3 pockets may highlight additional areas that are important for protein regulation and exploitable for drug targeting and understanding disease progression. There is a tight clustering of mutations in the BC loop of STAT3 on the periphery of the pY pocket. Similar to the dimerization interface, mutations at these residues can either enhance or reduce STAT activity. This region is in close proximity to the pY pocket, pY+3 pocket, dimerization interface, and STAT linker domain and likely serves as an important allosteric communication bridge for interdomain signaling. As such, interactions at this region of the BC loop require a complex balance of flexibility and rigidity. For instance, at the STAT3 BC3 position, a mutation at Ser614Arg [39,40,41,42] leads to hyperactivation and LGLL, whereas a Ser614Gly [34] results in LOF and AD-HIES. Increasing the positive electrostatic potential at this region generally leads to STAT hyperactivation and draws the BC loop closer into the pY pocket. Mutations found at neighboring sites BC5, BC6, and B7 delineate similar trends, where Glu616Lys [45], Glu616Gly [44], Gly617Arg [44], and Gly618Arg [47,48] are found in diffuse large B-cell lymphoma and NK-malignancies. Corresponding electronegative or bulky substitutions are associated with AD-HIES and dysfunctional STAT3 (Gly617Glu [46], Gly618Asp [35,37], and Gly617Val [34]).

There are additional mutations that are located across STAT5 and identified in single patient cases. Given the general robust nature of STAT5 to mutations, it is difficult to assess the oncogenic driving capacity of a single mutation, or whether the disease is multifactorial in etiology. For instance, a mutation in the short βA strand was identified within STAT5B as Gly596Val [79]. However, this mutation was identified in a chimeric protein of STAT5B and retinoic acid receptor-α (STAT5B-RARα), which is associated with acute promyelocytic leukemia (APL) and is also resistant to all-trans retinoic acid therapy. All-trans retinoic acid (ATRA) is capable of inducing remission in almost all APL cases, with several exceptions [125]. Since this is a rare subtype of APL, where the fusion protein was identified in a small minority of cases (<10), it is difficult to judge the importance of the mutation to the progression of the disease, although the residue is conserved across species. Notably, a STAT3-RARα fusion was also recently discovered in an APL patient with a similar ATRA-resistance profile [126]. Surprisingly, no STAT5A fusions have been discovered. It would be interesting to define if the dimerization mechanism of these RARα fusions is mediated through a partially intact SH2 domain or other dimerization domain of STAT. Other mutations in STAT5B are located in non-hotspots including Asp634Val (βC8), Gln636Pro (CD2), and Arg659Cys (αB4) and were identified in patients with T-PLL and MEITL. Similar to STAT3, these mutations are in close proximity to the dimerization interface and increase the hydrophobicity of the region, which can facilitate hyperactivation. It is interesting to note that T-PLL represents the disease with most hyperactive STAT3/5B and JAK3 mutations among all subgroups of largely untargeted orphan T-cell neoplasias, and future targeting efforts in this pathway will likely benefit these patients.

## 4. Conclusions

Disease associated mutations are more frequently identified in STAT3 compared to STAT5B, suggesting that STAT5B is more robust to the alterations in structural motifs, or that STAT3 has a more pronounced role in normal physiological functioning. However, it is clear that even slight alterations to electronics or sterics in the SH2 domain can dramatically alter STAT3 activity. In STAT3, the majority of mutations identified in the pY pocket impair protein function, with the most substantial effects observed upon mutation of conserved Sheinerman residues (Figure 1f). In STAT5B, pY mutations are generally activating with the Asn642His substitution occurring most frequently in aggressive T-cell cancers (Figure 1g). The pY+3 specificity pockets are characterized by multiple mutations with variable effects. Broadly, mutations that improve hydrophobicity or introduce aromaticity lead to hyperactivation, while increases in pY+3 pocket polarity or removal of aromatic substituents diminish STAT function. This trend is also observed at the SH2 domain dimerization interface which is a hot-spot for mutations, and different substitutions at a single position can result in severe loss- or gain-of-function. Finally, the BC loop may be a critical region for allosteric communication pathways throughout the protein and has been evolutionarily tuned for the precise interactions. As such, marginally reducing electronegativity or increasing electropositivity leads to hyper- and hypo-activation, respectively.

Currently, additional structural studies and molecular dynamics simulations are required for a better understanding of the molecular mechanisms of STAT3/5B mutations at different sites within the SH2 domain. Considering the conformational flexibility of the main binding sites with state-of-the-art computational methods, for example thermodynamic integration, should be more thoroughly exploited in further work. These can be used to propose alternative treatments or highlight therapeutic approaches. For instance, the relative instability of the wild type STAT3 protein is shown to be amplified by AD-HIES-causing mutations. The use of small molecules that can trigger stimulation of protein chaperones to rescue dysfunctional STAT3 mutants has been shown to be effective in cellulo [122]. Alternatively, hyperactivated STAT3 is only marginally more stable than the wild-type protein which may be exploited by degradation enhancing therapeutic strategies such as the use of PROTACs and hydrophobic tagging. These efforts can be extended to STAT5 as well as examining the Asn642His site, as this hot-spot mutation mimics SH2 domain superbinders and is excessively aggressive due to its prime role in the pY pocket. Collectively, these structural studies offer a predictive approach for understanding the molecular foundations of additional mutations identified in the SH2 domain, based on their location and alterations to pocket electronics and sterics.

## Figures and Tables

**Table 1:** Disease-associated mutations in the STAT3 SH2 domain.

Mutation	Position	Location	Residue Relevance	Nucleotide Substitution	Cases	Pathology	Type	Ref
K591E	αA2	pY	Sheinerman	1771A>G	1	AD-HIES	Germline	[30]
K591M	αA2	pY	Sheinerman	1772A>T	1	AD-HIES	Germline	[31]
R593P	αA4	pY	-	1778G>C	1	AD-HIES	Germline	[32]
R609G	βB5	pY	Sheinerman& Signature	1825A>G	1	AD-HIES	Germline	[33]
S611G	βB7	pY	Sheinerman	1831A>G	2	AD-HIES	Germline	[34,35]
S611N	βB7	pY	& Signature Sheinerman	1832G>A	2	AD-HIES	Germline	[36,37]
S611I	βB7	pY	Sheinerman & Signature	1832G>T	1	AD-HIES	Germline	[38]
S614G	BC3	pY	Sheinerman	18040A>G	1	AD-HIES	Germline	[34]
S614R	BC3	pY	Sheinerman	1842C>G	1	T-LGLL	Somatic	[39,40,41,42,43]
					2	NK-LGLL		
					1	ALK^-^ALCL		
					1	HSTL		
E616G	BC5	pY	BC loop	1847A>G	1	DLBCL, NOS	Somatic	[44]
E616K	BC5	pY	BC loop	1846G>A	1	NKTL	Somatic	[45]
G617E	BC6	pY	BC loop	1850G>A	1	AD-HIES	Germline	[46]
G617V	BC6	pY	BC loop	1850G>T	1	AD-HIES	Germline	[34]
G617R	BC6	pY	BC loop	1849G>A	1	DLBCL, NOS	Somatic	[44]
G618R	BC6	pY	BC loop	1852G>C	1	ALK^-^ALCL	Somatic	[47,48]
					1	T-LGLL		
G618D	BC6	pY	BC loop	1853G>A	2	AD-HIES	Germline	[35,37]
T620A	βC2	pY	-	1858A>G	2	AD-HIES	Germline	[34,49]
T620S	βC2	pY	-	1859C>G	1	AD-HIES	Germline	[33]
F621V	βC3	pY+3	Hydro. Sys.	1861T>G	3	AD-HIES	Germline	[36,37,50]
F621L	βC3	pY+3	Hydro. Sys.	1863C>G	2	AD-HIES	Germline	[51,52]
F621S	βC3	pY+3	Hydro. Sys.	1862T>C	1	AD-HIES	Germline	[53]
T622I	βC4	pY	-	1865C>T	4	AD-HIES	Germline	[30,34,36]
D627E	CD1		-	1881C>A	1	ATLL	Somatic	[54]
S636F	βD4	pY	Sheinerman	1907C>T,	1	AD-HIES	Germline	[49]
S636Y	βD4	pY	Sheinerman	1907C>A	1	AD-HIES	Germline	[30]
V637M	βD5	pY+3	Sel. Filter	1909G>A	43	AD-HIES	Germline	[30,32,33,34,35,36,37,49,52,55,56]
V637L	βD5	pY+3	Sel. Filter	1909G>T	1	AD-HIES	Germline	[36]
V637A	βD5	pY+3	Sel. Filter	1910T>C	1	AD-HIES	Germline	[30]
E638G	βD6	pY	Sheinerman	1913A>G	4	AD-HIES	Germline	[49,51,57,58]
P639A	βD7	pY+3	-	1915C>G	1	AD-HIES	Germline	[37]
P639S	βD7	pY+3	-	1915C>T*	2	AD-HIES	Germline	[30,34]
P639T	βD7	pY+3	-	1915C>A	1	AD-HIES	Germline	[35]
Y640F	DB’1	pY+3	-	1919A>T	56	T-LGLL	Somatic	[43,44,47,48,57,59,60,61,62,63,64,65,66,67,68,69]
					2	IHT		
					3	CLPD-NKs		
					2	NK-LGLL		
					1	DLBCL, NOS		
					1	ANKL		
					1	NKTL		
					1	Sezary		
					3	HSTL		
K642E	αB’1	-	Dimer Inter	1924A>G	1	AD-HIES	Germline	[34]
Q643K	αB’2	-	Dimer Inter	1927C>A	1	T-LGLL	Somatic	[68]
Q644P	αB’3	-	Dimer Inter	1929A>C	3	AD-HIES	Germline	[52,70]
Q644del	αB’3	-	Dimer Inter	1930del CAG	2	AD-HIES	Germline	[35,36]
N646K	αB’5	pY+3	Dimer Inter	1938C>G	2	EOAD	Germline	[71]
N647D	αB’6	pY+3	Dimer Inter	1939A>G	8	AD-HIES	Germline	[36,72]
N647I	αB’6	pY+3	Dimer Inter	1940A>T	3	CLPD-NK	Somatic	[43,57,65,67]
					6	T-LGLL		
					1	HSTL		
E652K	αB3	pY+3	Dimer Inter	1954G>A	1	AD-HIES	Germline	[36]
G656D	αB7	pY+3	Dimer Inter	1967G>A	1	T-LGLL	Somatic	[73]
G656_Y657insF	αB7	pY+3	Hydro. Sys.	1968C>T; 1969_1970insTTT	1	IHCA	Somatic	[66]
Y657C	BC1*	pY+3	Hydro. Sys.	1970A>G	5	AD-HIES	Germline	[30,34,36,58]
Y657S	BC1*	pY+3	Hydro. Sys.	1970A>C	1	AD-HIES	Germline	[74]
Y657N	BC1*	pY+3	Hydro. Sys.	1969T>A	1	AD-HIES	Germline	[52]
Y657ins	BC1*	pY+3	Hydro. Sys.	-	1	TCL	Somatic	[47]
Y657dup	BC1*	pY+3	Hydro. Sys.	-	3	T-LGLL	Somatic	[48,65,72]
Y657_M660dup	BC1*	pY+3	Hydro. Sys.	1969T_1980G dup	1	IHCA	Somatic	[66]
K658M	BC2*	pY+3	Dimer Inter	1973A>T	2	T-LGLL	Somatic	[67,72]
K658N	BC2*	pY+3	Dimer Inter	1974G>T	1	T-LGLL EOAD	Somatic/Germline	[65,71]
K658Y	BC2*	pY+3	Dimer Inter	1972A>T; 1974G>T	1	IHCA	Somatic	[66]
K658E	BC2*	pY+3	Dimer Inter	1972A>G	1	AD-HIES	Germline	[75]
I659N	BC3*	pY+3	Hydro. Sys.	1976A>T	1	AD-HIES	Germline	[58]
I659L	BC3*	pY+3	Hydro. Sys.	1975A>C	2	T-LGLL	Somatic	[57,72]
M660R	BC4*	pY+3	Dimer Inter	1979T>G	1	AD-HIES	Germline	[55]
M660T	BC4*	pY+3	Dimer Inter	1978T>A	1	AD-HIES	Germline	[76]
D661I	BC5*	-	Dimer Inter	1981G>A; 1982A>T	1	CLPD-NK	Somatic	[67]
D661Y	BC5*	-	Dimer Inter	1981G>T	1	NK-LGL	Somatic	[41,47,48,60,63,65,67,68,72,77]
					56	T-LGL		
					1	HSTL		
					10	NKTL		
D661ins	BC5*	-	Dimer Inter	-	1	T-LGLL	Somatic	[47]
D661V	BC5*	-	Dimer Inter	1981A>T	10	T-LGLL	Somatic	[65,67]
D661H	BC5*	-	Dimer Inter	1981G>C	1	T-LGLL	Somatic	[65]
A662V	BC6*	-	Dimer Inter	1985C>T	1	ALK^-^ALCL	Somatic	[78]
T663I	BC7*	-	Dimer Inter	1988, 1989>TT	2	DLBCL/B1	Germline	[44,75]
I665N	BC9*	-	Dimer Inter	1998T>A	2	AD-HIES	Germline	[34]
V667L	BC11*	-	Dimer Inter	1999C>G	1	NKTL	Somatic	[45]
S668F	BC12*	-	Dimer Inter	2003C>T	3	AD-HIES	Germline	[30,34,37]
S668Y	BC12*	-	Dimer Inter	2003C>A	1	AD-HIES	Germline	[34]

The final search date for mutations from medical case reports and literature was 30 August, 2019. Abbreviations: AD-HIES, autosomal-dominant Hyper IgE syndrome; ALK^-^ALCL, anaplastic lymphoma kinase negative anaplastic large cell lymphoma; ANKL, aggressive natural killer cell leukemia; ATLL, adult T-cell leukemia lymphoma; EOAD, early onset autoimmune disease; CLPD-NKs, Chronic lymphoproliferative disorders of natural killer cells; DLBCL, NOS, Diffuse large B-cell lymphoma, not-otherwise-specified; NKTL, extranodal NK/T-cell lymphoma; HSTL, Hepatosplenic T-cell lymphoma; IHAC, inflammatory hepatocellular adenomas; IHT, inflammatory hepatocellular tumors; NK-LGLL, Natural killer cell large granular lymphocytic leukemia; T-LGLL, T-cell large granular lymphocytic leukemia; TCL, γδ-T-cell lymphoma.

**Table 2:** Disease-associated mutations in the STAT5B SH2 domain.

Mutation	Position	Location	Residue Relevance	Nucleotide Substitution	Cases	Pathology	Type	Ref.
G596V	βA2	-	-	1787G>T	1	APL	Somatic	[79]
T628S	βC2	pY	-	1883C>G	11	T-PLL	Somatic	[43,80,81]
					1	MEITL		
					3	HSTL		
					1	Eosinophilia		
A630P	βC4	pY	-	1888G>C	1	GHI	Germline *	[82]
D634V	βC8	pY	-	1901A>T	1	T-PLL	Somatic	[83]
Q636P	CD2	pY+3	-	1907A>C	1	MEITL	Somatic	[81]
N642H	βD4	pY	Sheinerman	1924A>C	39	MEITL	Somatic	[41,62,69,77,80,81,83,84,85,86,87,88,89,90,91,92,93,94,95,96,97,98,99,100,101,102]
					33	T-PLL		
					29	Eosinophilia		
					28	T-ALL		
					7	HSTL		
					11	LGLL		
					3	PCTL		
					3	Sézary		
					1	AAA		
					1	CNL		
					1	PTCL, NOS		
					1	AML		
F646S	DB’1	pY+3	-	1937T>C	1	GHI	Germline *	[103]
T648S	DB’3	pY+3	-	1942A>T	1	T-ALL	Somatic	[101]
R659C	αB’4	Dimer Inter	-	1975C>T	1	T-PLL	Somatic	[80]
Y665F	BC3*	Dimer Inter	Hydro. Sys.	1994A>T	6	T-PLL	Somatic	[43,80,101]
					3	HSTL		
					2	T-ALL		
					2	NKTL		
					5	LGLL		
Y665H	BC3*	Dimer Inter	Hydro. Sys.	1993T>C	2	T-PLL	Somatic	[80]

* Patients were homozygous for the point mutation. The final search date for mutations from medical case reports and literature was 30 August, 2019. Abbreviations: AAA, acquired aplastic anemia; AML, acute myeloid leukemia; APL, acute promyelocytic leukemia; CNL, chronic neutrophilic leukemia; GHI, growth hormone insensitivity; HSTL, hepatosplenic T-cell lymphoma; MEITL, monomorphic epitheliotropic intestinal T cell lymphoma; PCTL, primary cutaneous γδ T-cell lymphoma; PTCL-NOS, peripheral T-cell lymphoma not-other-specified; T-ALL, T-cell acute lymphoblastic leukemia; T-LGLL, T-cell large granular lymphocytic leukemia; T-PLL, T-cell prolymphocytic leukemia.
